# Primary angiosarcoma arising in an angiomyolipoma of the kidney: case report and literature review

**DOI:** 10.1186/s13000-018-0730-z

**Published:** 2018-08-16

**Authors:** Hongwei Guan, Lizhi Zhang, Qiuping Zhang, Wenjing Qi, Suling Xie, Jinping Hou, Huali Wang

**Affiliations:** Department of Pathology, Dalian Medical University First Hospital, 222 Zhongshan Road, Xigang District, Dalian, 116011 Liaoning China

**Keywords:** Primary angiosarcoma, Angiomyolipoma, Kidney, Pathology

## Abstract

**Background:**

Primary angiosarcoma of the kidney is a rare and aggressive malignant tumor presenting with a recognizable vascular differentiation. Its prognosis is fatal and the pathogenesis remains unclear. Renal angiomyolipoma is a relatively infrequent renal cortical neoplasm and is composed of variable proportions of adipose tissue, spindle cells, epithelioid smooth muscle cells and abnormal thick-walled blood vessels.

**Case presentation:**

Here, we reported a case in which a 64-year-old woman presenting with the chief complaint of a progressively enlarged mass in the left abdomen. Abdominal computed tomography confirmed presence of a tumor measuring 18 cm × 11 cm in the left posterior renal fascia. Microscopic examination and immunohistochemical staining confirmed co-existence of angiomyolipoma and angiosarcoma. Furthermore, the two components interspersed with each other and there were transitional zones between them.

**Conclusions:**

In this case, we described for the first time a primary renal angiosarcoma possibly arising in a pre-existing angiomyolipoma of the kidney.

## Background

Angiosarcoma (AS) is a rare and aggressive malignant tumor presenting with a recognizable vascular differentiation. It occurs mainly in the adulthood and elderly, with occasional cases reported in children [1]. Angiosarcoma is usually located in the skin, superficial soft tissue or other organs, such as bone, breast or liver [2]. Primary renal angiosarcoma is extremely rare and its prognosis is poor due to the rapid local recurrence and bloodstream dissemination [3].

Renal angiomyolipoma (AML) is the most common mesenchymal tumor of the kidney and it was first reported by Morgan in 1951 [4]. The World Health Organization (WHO) defines renal angiomyolipoma as a benign mesenchymal tumor composed of variable proportions of adipose tissue, spindle cells, epithelioid smooth muscle cells and abnormal thick-walled blood vessels [5]. It belongs to a family of lesions called perivascular epithelioid cell tumors (PEComas), which are characterized by proliferation of perivascular epithelioid cells [5]. They are not always located in the kidney and also can be found in uterus, liver, fallopian tubes and spleen [6].

Here we described a case of primary renal angiosarcoma concomitant with an AML in a 64-year-old woman. To our best knowledge, this is the first report of a renal angiosarcoma arising in an AML background.

## Case presentation

**Fig. 1:**
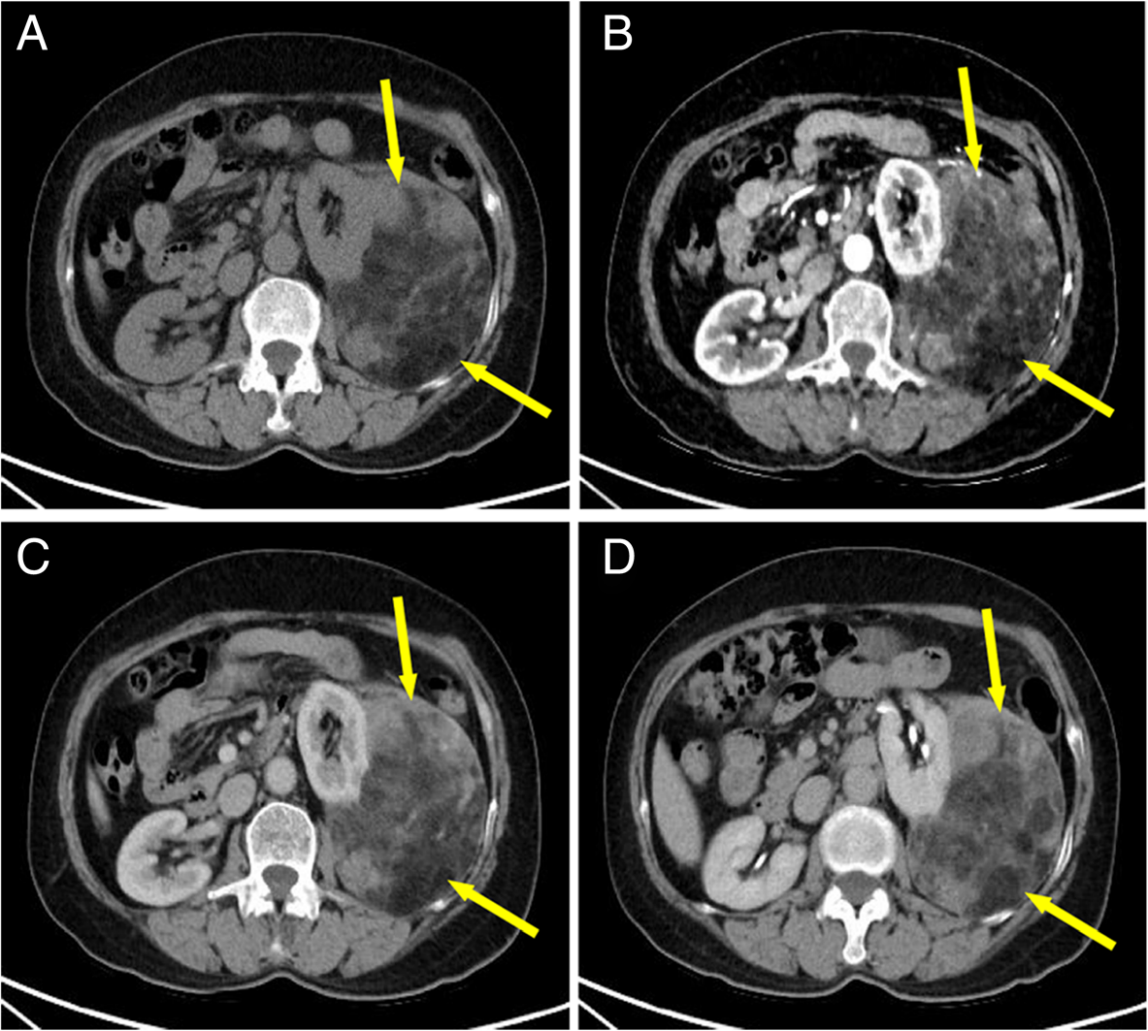
The complex lesion of the kidney on computed tomography (CT). **a**. Plain CT image showed a large mixed density mass in the left renal fascia, which was closely related to the left kidney (yellow arrows). **b**. Dynamic contrast CT (arterial phase) showed the mass was obviously uneven, and small vessels could be seen around the lesion (yellow arrows). **c**. Dynamic contrast CT (venous phase) showed the enhancement degree of the mass decreased compared to that in the arterial phase (yellow arrows). **d**. Dynamic contrast CT (equilibrium phase) showed the degree of enhancement was similar to that of venous phase (yellow arrows)

A 64-year-old woman presented to our hospital with the chief complaint of a progressively enlarged mass in the left abdomen accompanied with flank pain, fatigue and weight loss for the last few months. Physical examination revealed a well-defined mass on the left abdomen. Routine blood tests showed that hemoglobin was 111 g/L (normal range was 115 g/L to 150 g/L) and hematocrit was 34% (ranging 35 to 45%), indicating anemia. The remaining blood tests were normal. Abdominal computed tomography (CT) confirmed presence of a tumor measuring 18 cm × 11 cm on the left posterior perinephric capsule (Fig. 1a), with enhanced density after administration of contrast medium (Fig. 1b-d).

**Fig. 2:**
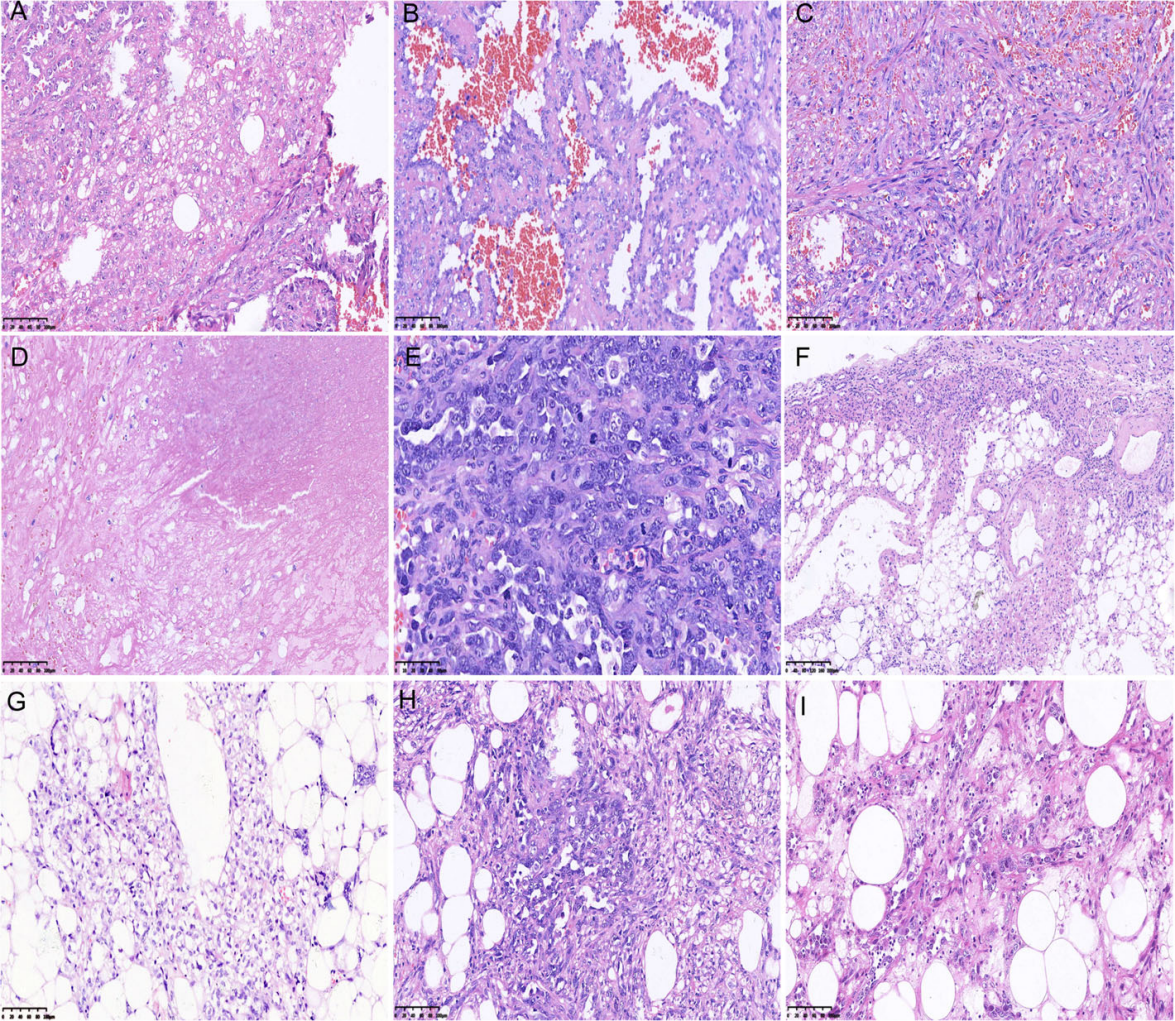
Angiosarcoma (AS) and angiomyolipoma (AML) in the renal cortex. **a**. The transitional zones between AS and AML (× 200); **b**. The well-differentiated areas of AS (× 200); **c**. The poorly-differentiated areas of AS (× 200); **d**. Tumorous necrosis in the lesion (× 200); **e**. Mitotic figures per high-power field (× 400); **f** (× 100) and **g** (× 200). AML regions in the lesion; **h** and **i**. Atypical cells with the morphological and structural characteristics resembling AS in areas of AML (× 200)

The mass was excised and a pathologic consultation was requested. On macroscopic examination, the specimen was composed of a giant nodular mass measuring 18 cm × 11 cm × 9 cm with a little attached portion of normal renal cortex. The outer surface of the mass was smooth and brownish in color. Sectioning revealed patchy areas of hemorrhage and necrosis. Microscopic examination was performed on paraffin-embedded sections stained with hematoxylin and eosin. Histopathological examination revealed that two components interspersed with each other within this tumor and there were transitional zones between the two (Fig. 2a). One component comprised of atypical cells arranged in multiple architectures. Some well-differentiated fields were composed of irregular interanastomosing vascular spaces or channels lined with discrete and large endothelial cells with variable degrees of cytological pleomorphism, nuclear atypia and multilayering (Fig. 2b). Many of these structures contained red blood cells, indicating a vascular lesion. Some poorly-differentiated fields were composed predominantly of atypical epithelioid cells arranged in solid areas (Fig. 2c). The epithelioid cells had irregular round or oval nuclei with prominent nucleoli, abundant cytoplasm and a high degree of nuclear atypia. Tumorous necrosis was obvious (Fig. 2d), and up to five mitotic figures per high-power field were observed (Fig. 2e). The other component (including the areas adjacent to the renal cortex) was primarily composed of adipose tissue, smooth muscle cells and abnormal thick-walled blood vessels (Fig. 2f and g), indicating the feature of AML. But in multifocal areas of AML, atypical cells with the morphological and structural characteristics resembling those of the first component were observed (Fig. 2h and i).

**Fig. 3:**
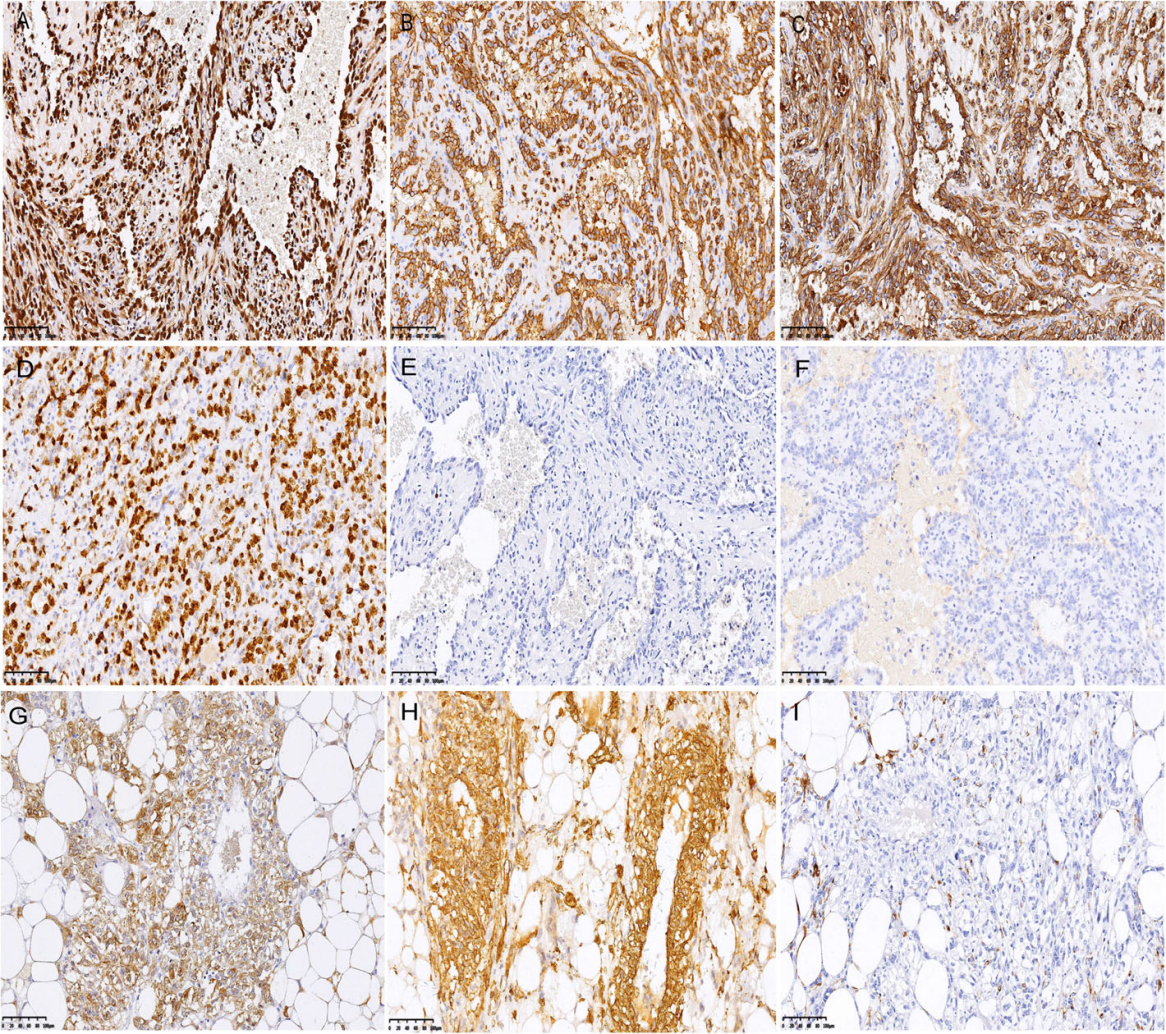
Different expressions of ERG (**a**), CD31 (**b**), CD34 (**c**), Ki-67 (**d**), HMB45 (**e**), Melan-A (**f**) in AS (paired with H&E in Fig. 2b) and Melan-A (**g**), SMA (**h**) and HMB45 (**i**) in AML (paired with H&E in Fig. 2**g**) (× 200)

**Fig. 4:**
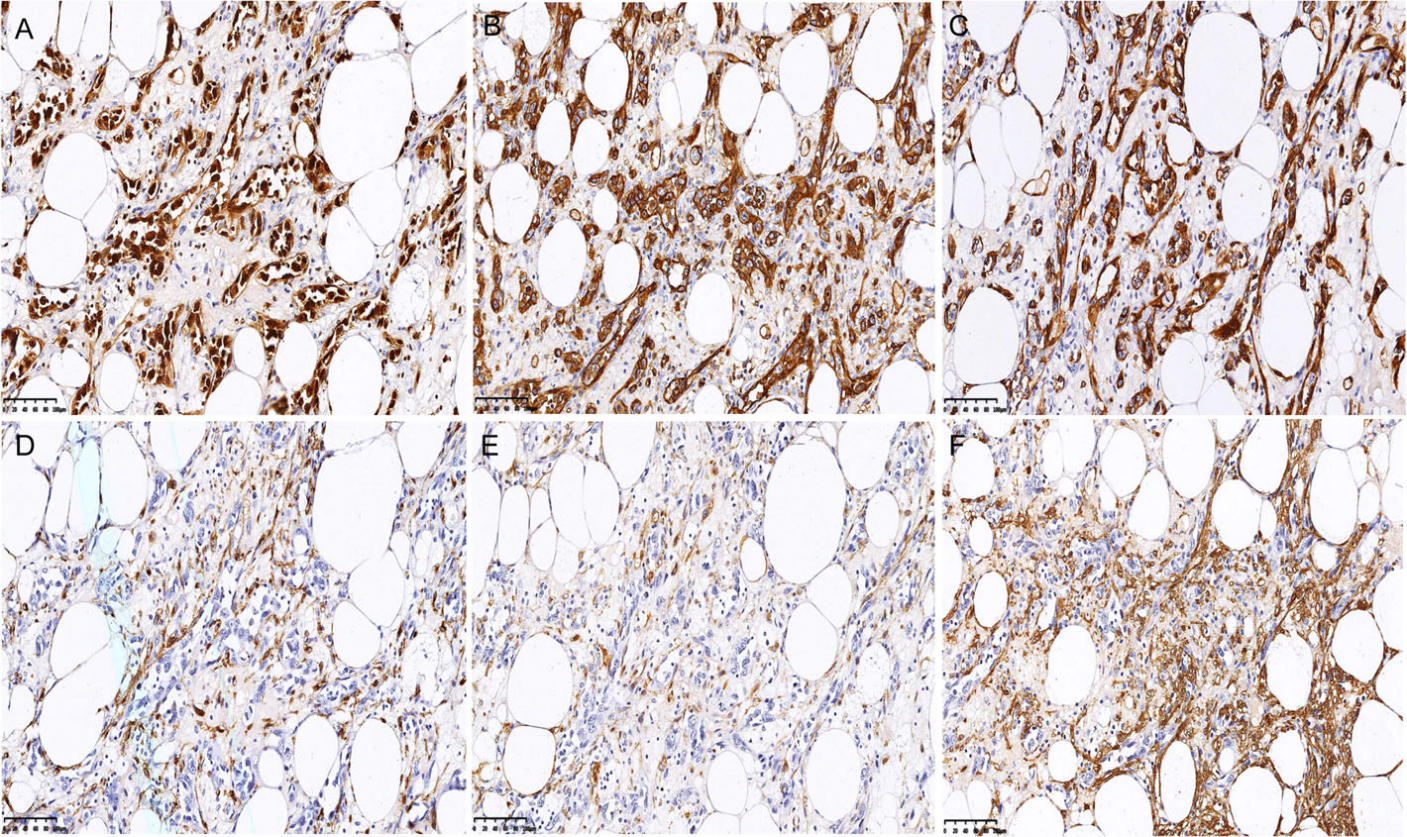
Different expressions of ERG (**a**), CD31 (**b**), CD34 (**c**), HMB45 (**d**), Melan-A (**e**) and SMA (**f**) in the atypical cells with the morphological and structural characteristics resembling AS in areas of AML (paired with H&E in Fig. 2i) (× 200)

An immunohistochemical study was performed on formalin-fixed paraffin-embedded tissue block to define the histogenesis of the lesion. Several pre-diluted antibodies against ERG, CD31, CD34, Ki-67, S-100, HMB45, Melan A, SMA, Actin, Vimentin, CD10, EMA, PAX-8, RCC and pan-cytokeratin were used. The results showed that the atypical cells in the areas of the first component were reactive strongly and diffusely to ERG (Fig. 3a), CD31 (Fig. 3b), CD34 (Fig. 3c), and Vimentin, but negative to HMB45 (Fig. 3e), Melan-A (Fig. 3f), SMA, Actin and S-100. In addition, the cells were also negative to pan-cytokeratin, PAX-8, RCC, EMA and CD10. The cell proliferation marker Ki-67 (MIB-1) was positive in about 50% of the atypical cells (Fig. 3d). To sum up, the immunohistochemical results supported the diagnosis of angiosarcoma. On the contrary, the cells in the areas of AML were reactive strongly and diffusely to Melan-A (Fig. 3g) and SMA (Fig. 3h), focally to HMB45 (Fig. 3i) and Actin, confirming the diagnosis of AML. However, the atypical cells in the AML background were positive to ERG (Fig. 4a), CD31 (Fig. 4b), CD34 (Fig. 4c), but negative to HMB45 (Fig. 4d), Melan-A (Fig. 4e) and SMA (Fig. 4f). On the basis of clinical, histologic and immunohistochemical findings, a final diagnosis of renal angiosarcoma concomitant with an AML was made.

Three months after the surgery, the patient presented again with progressive abdominal distention for about one week. CT examination showed diffuse thickening and nodular deposits of omentum in the left lower abdomen and a lot of abdominal and pelvic effusion. Combined with the history and imaging examinations, extensive abdominal metastasis was highly suspicious. The patient received intraperitoneal cisplatin perfusion and symptomatic treatments such as paracentesis and analgesia. However, one month later, she died.

## Discussion

Primary renal angiosarcoma is a rare malignant vascular tumor originating from endothelial cells. It has an extremely high mortality rate due to rapid growth and distant metastasis at the time of diagnosis or shortly afterwards. Angiosarcoma is considered to originate from endothelial cells in tissues or from circulating stem cells which are recruited from bone marrow or locations of extra-medullary hematopoiesis [7, 8]. Gene amplifications or genetic mutations for *p53, VEGF, Ras, Myc* and *MDM2* have been explored in a great deal of angiosarcoma tissues, but the genetic changes found are complicated [9]. Although the exact etiology is unknown, many different environmental factors are associated with the occurrence of angiosarcoma. These factors include exposure to arsenic, vinyl chloride, thorium dioxide, radiotherapy and chronic lymphedema of any causes [2, 10–14].The diagnosis of this lesion is extremely difficult if not impossible if only the clinical and radiological features are considered. Therefore, the importance of post-operative pathological examination cannot be ignored. Studies showed that angiosarcoma could occur on the background of other tumors, such as schwannoma [15, 16], hemangioma and lymphangioma [17, 18], malignant germ cell tumor [19], leiomyoma [20] and tuberous sclerosis complex (TSC)-associated lymphangioleiomyomatosis (LAM) [21], all of which were very rare.

Renal AMLs are uncommon neoplasms accounting for less than 1% of surgically removed tumors [22]. And they are often associated with tuberous sclerosis complex (TSC), which is an autosomal dominant genetic disease because of losses of *TSC1* (9q34) or *TSC2* (16p13.3) genes [23, 24]. In recent years, great advances have been made in understanding TSC and related lesions. In particular, *TSC* genes seem to play an important role in regulating mTOR pathway [25]. However, most AMLs occur sporadically [22]. Similar changes of the *TSC* genes have been found in both TSC-related AMLs and sporadic cases. Kenerson et al. [26] have recently demonstrated that mTOR activity is increased in sporadic, non-TSC-related AMLs. In patients with TSC, AMLs are found predominantly in 30 to 40 years old women and they tend to be asymptomatic, small, multifocal, and bilateral. On the other hand, sporadic AMLs are seen in 40 to 70 years old women and are usually larger, symptomatic, single, and unilateral [27].

We described here, for the first time, a patient who had renal angiosarcoma concomitant with an AML verified by immunohistochemical staining. In this case, there was no definite boundary between the two components. On the contrary, they were interspersed with each other and there were transitional zones between them. In this context, the possibility of a collision tumor could be ruled out. Furthermore, in several areas of AML, we could find multifocal atypical cells with the morphological and structural characteristics resembling those of angiosarcoma. Immunohistochemical staining verified their vascular origin. Taking into account the above mentioned findings, we inferred that in this case, angiosarcoma might occur in a pre-existent AML. However, the etiology and pathogenesis are still very speculative. Explanations might be as follows. Firstly, angiosarcoma cells might originate from vascular endothelial cells in AML lesions. Previous studies have suggested a crucial role of the mTOR pathway in the malignant transformation of endothelial cells. Besides, Italiano et al. [28, 29] reported in their studies that mTOR pathway was activated in a subset of angiosarcomas. Considering the role of mTOR pathway in the pathogenesis of AML, we are more inclined to this possibility. However, we have no direct evidence. Secondly, angiosarcoma cells might have originated from lymphatic endothelial cells in AML lesions. Like Stewart-Treves syndrome, long-term postmastectomy lymphedema and regional immune deficiency are considered to be an important factor in the complication of angiosarcoma. Hayashi et al. [21] speculated in their report that lymphatic endothelial cells might have undergone persistent genetic alterations of *TSC2* and *TP53*, which eventually lead to malignant transformation toward angiosarcoma. Thirdly, angiosarcoma cells might have been derived from AML cells as a malignant transformation. However, in this case the angiosarcoma cells expressed neither Melan-A nor HMB45, and the immunoprofile of angiosarcoma clearly differed from that of renal AML. Even so, we cannot exclude the possibility that the renal angiosarcoma is simply a de novo occurrence other than associated with the AML lesion. However, more studies were needed to investigate the correlation between angiosarcoma and PEComas.

The prognosis of renal angiosarcoma is deadly because of local recurrence and extensive metastasis. At present, there appears to be no standard therapy because of the rarity of this malignancy. Most patients were treated with nephrectomy combined with radiotherapy, chemotherapy or recombinant interleukin-2 therapy [2, 30]. The optimal treatment still remains controversial. However, surgery seems to be the most effective treatment. In this case, the patient only received partial nephrectomy combined with intraperitoneal cisplatin perfusion and symptomatic treatment. Nevertheless, she died four months after the surgery, confirming the fatal prognosis of renal angiosarcoma.

## Conclusions

In conclusion, we described here, for the first time, a primary renal angiosarcoma possibly arising in an AML, and the AML might be the precursor of the primary angiosarcoma of the kidney. However, more studies are needed to detect the correlation between the two.

## Abbreviations

AML: Angiomyolipoma
AS: Angiosarcoma
CT: Computed tomography
LAM: Lymphangioleiomyomatosis
PEComa: Perivascular epithelioid cell tumor
TSC: Tuberous sclerosis complex

## References

[1] Deyrup AT, Miettinen M, North PE, Khoury JD, Tighiouart M, Spunt SL, Parham D, Weiss SW, Shehata BM (2009). Angiosarcomas arising in the viscera and soft tissue of children and young adults: a clinicopathologic study of 15 cases. Am J Surg Pathol.

[2] Omiyale AO (2015). Clinicopathological features of primary angiosarcoma of the kidney: a review of 62 cases. Transl Androl Urol.

[3] Zenico T, Saccomanni M, Salomone U, Bercovich E (2011). Primary renal angiosarcoma: case report and review of world literature. Tumori.

[4] Martignoni G, Pea M, Reghellin D, Zamboni G, Bonetti F (2008). PEComas: the past, the present and the future. Virchows Arch.

[5] Moch H, Humphrey PA, Ulbright TM, Reuter VE. WHO Classification of Tumours of the Urinary System and Male Genital Organs. LARC, Lyon. 2016; p. 62–64. 10.1016/j.eururo.2016.02.028 26996659

[6] Zapardiel I, Delafuente-Valero J, Bajo-Arenas JM (2011). Renal angiomyolipoma during pregnancy: review of the literature. Gynecol Obstet Investig.

[7] Young RJ, Brown NJ, Reed MW, Hughes D, Woll PJ (2010). Angiosarcoma. Lancet Oncol.

[8] Cohen SM, Storer RD, Criswell KA, Doerrer NG, Dellarco VL, Pegg DG, Wojcinski ZW, Malarkey DE, Jacobs AC, Klaunig JE (2009). Hemangiosarcoma in rodents: mode-of-action evaluation and human relevance. Toxicol Sci.

[9] Yang J, Kantrow S, Sai J, Hawkins OE, Boothby M, Ayers GD, Young ED, Demicco EG, Lazar AJ, Lev D, Richmond A (2012). INK4a/ARF [corrected] inactivation with activation of the NF-kappaB/IL-6 pathway is sufficient to drive the development and growth of angiosarcoma. Cancer Res.

[10] Penel N, Marreaud S, Robin YM, Hohenberger P (2011). Angiosarcoma: state of the art and perspectives. Crit Rev Oncol Hematol.

[11] Brown JG, Folpe AL, Rao P, Lazar AJ, Paner GP, Gupta R, Parakh R, Cheville JC, Amin MB (2010). Primary vascular tumors and tumor-like lesions of the kidney: a clinicopathologic analysis of 25 cases. Am J Surg Pathol.

[12] Papadimitriou VD, Stamatiou KN, Takos DM, Adamopoulos VM, Heretis IE, Sofras FA (2009). Angiosarcoma of kidney: a case report and review of literature. Urol J.

[13] Akkad T, Tsankov A, Pelzer A, Peschel R, Bartsch G, Steiner H (2006). Early diagnosis and straight forward surgery of an asymptomatic primary angiosarcoma of the kidney led to long-term survival. Int J Urol.

[14] Popper H, Thomas LB, Telles NC, Falk H, Selikoff IJ (1978). Development of hepatic angiosarcoma in man induced by vinyl chloride, thorotrast, and arsenic. Comparison with cases of unknown etiology. Am J Pathol.

[15] Trassard M, Le Doussal V, Bui BN, Coindre JM (1996). Angiosarcoma arising in a solitary schwannoma (neurilemoma) of the sciatic nerve. Am J Surg Pathol.

[16] Brown RW, Tornos C, Evans HL (1992). Angiosarcoma arising from malignant schwannoma in a patient with neurofibromatosis. Cancer.

[17] Meis-Kindblom JM, Kindblom LG (1998). Angiosarcoma of soft tissue: a study of 80 cases. Am J Surg Pathol.

[18] Fletcher CD, Beham A, Bekir S, Clarke AM, Marley NJ (1991). Epithelioid angiosarcoma of deep soft tissue: a distinctive tumor readily mistaken for an epithelial neoplasm. Am J Surg Pathol.

[19] Ulbright TM, Clark SA, Einhorn LH (1985). Angiosarcoma associated with germ cell tumors. Hum Pathol.

[20] Tallini G, Price FV, Carcangiu ML (1993). Epithelioid angiosarcoma arising in uterine leiomyomas. Am J Clin Pathol..

[21] Hayashi T, Koike K, Kumasaka T, Saito T, Mitani K, Terao Y, Ogishima D, Yao T, Takeda S, Takahashi K, Seyama K (2012). Uterine angiosarcoma associated with lymphangioleiomyomatosis in a patient with tuberous sclerosis complex: an autopsy case report with immunohistochemical and genetic analysis. Hum Pathol.

[22] Pillay K, Lazarus J, Wainwright HC (2003). Association of angiomyolipoma and oncocytoma of the kidney: a case report and review of the literature. J Clin Pathol.

[23] van Slegtenhorst M, de Hoogt R, Hermans C, Nellist M, Janssen B, Verhoef S, Lindhout D, van den Ouweland A, Halley D, Young J (1997). Identification of the tuberous sclerosis gene TSC1 on chromosome 9q34. Science.

[24] European Chromosome 16 Tuberous Sclerosis Consortium (1993). Identification and characterization of the tuberous sclerosis gene on chromosome 16. Cell.

[25] Kwiatkowski DJ (2003). Tuberous sclerosis: from tubers to mTOR. Ann Hum Genet.

[26] Kenerson H, Folpe AL, Takayama TK, Yeung RS (2007). Activation of the mTOR pathway in sporadic angiomyolipomas and other perivascular epithelioid cell neoplasms. Hum Pathol.

[27] Tamboli P, Ro JY, Amin MB, Ligato S, Ayala AG (2000). Benign tumors and tumor-like lesions of the adult kidney. Part II: benign mesenchymal and mixed neoplasms, and tumor-like lesions. Adv Anat Pathol.

[28] Italiano A, Chen CL, Thomas R, Breen M, Bonnet F, Sevenet N, Longy M, Maki RG, Coindre JM, Antonescu CR (2012). Alterations of the p53 and PIK3CA/AKT/mTOR pathways in angiosarcomas: a pattern distinct from other sarcomas with complex genomics. Cancer.

[29] Lahat G, Dhuka AR, Hallevi H, Xiao L, Zou C, Smith KD, Phung TL, Pollock RE, Benjamin R, Hunt KK (2010). Angiosarcoma: clinical and molecular insights. Ann Surg.

[30] Iacovelli R, Orlando V, Palazzo A, Cortesi E (2014). Clinical and pathological features of primary renal angiosarcoma. Can Urol Assoc J.

